# Single type infection of human papillomavirus as a cause for high-grade cervical intraepithelial neoplasia and invasive cancer in Japan

**DOI:** 10.1016/j.pvr.2018.10.001

**Published:** 2018-10-26

**Authors:** Jinichi Sakamoto, Shoji Kamiura, Kaori Okayama, Mitsuaki Okodo, Takeo Shibata, Yasuhiro Osaka, Satoko Fujita, Emi Takata, Hiroaki Takagi, Masahiro Takakura, Toshiyuki Sasagawa

**Affiliations:** a Kanazawa Medical University, Daigaku 1-1, Uchinada-cho, Kahoku-gun, Ishikawa 920-0265, Japan; b Osaka International Cancer Institute, 3-1-69, Otemae, Chuo-ku, Osaka-shi, 541-8567 Osaka, Japan; c School of Medical Technology, Faculty of Health Science, Gumma Paz University, Gumma, Japan; d Department of Medical Technology, Faculty of Health Sciences, Kyorin University, 5-4-1 Shimorenjaku Mitaka, Tokyo 181-0013, Japan

## Abstract

To elucidate oncogenic human papilloma virus (HPV) types in Japan, HPV genotyping was performed in 1526 cervical intraepithelial neoplasia (CIN) and 371 invasive cervical cancer (ICC) patients with the novel Genosearch-31+5 HPV test. The HPV-positive rates were 89.3% and 90.8% in CIN and ICC. Regarding single-type infections, 13 internationally recognized high-risk (13HR) types excluding HPV 35, and probably HR HPV 53, 67, 69, and 70 were identified in ICC, suggesting that all these types may be oncogenic. HPV16 and 18 were identified in both SCC and adenocarcinoma (ADC). HPV HPV52, 31 and 58 (alpha-9) were predominantly detected in SCC, whereas HPV 18, 45, 39 and 59 (alpha-7) were in ADC. The prevalence of HPV 18 in SCC significantly decreased with increasing age of patients, whereas the opposite trend was observed in the other HR types. HPV18 is likely to induce SCC rapidly. All ICC cases aged 20–29 were positive for HPV 16 or 18, suggesting that present HPV 16, 18 vaccines may be quite effective to prevent ICC in young women.

## 1 Introduction

Cervical cancer is the third most common gynecologic cancer in developed countries [1], [2]. Cervical cancer screening was implemented in Japan in 1980. The incidence and mortality rates have decreased in older women, whereas they have increased in young women in recent years. The number of deaths by cervical cancer in Japan is estimated at 3500 women annually [3]. Approximately half of cervical cancer patients in Japan are women of reproductive age (younger than 50 years) [3]. Recently more than 200 women aged 20–39 years die every year. Cervical cancer is caused by human papillomavirus (HPV) infection, such as HPV 16 or 18 [4], [5]. Thus, cervical cancer is now a preventable disease through immunization with prophylactic HPV 16 and 18 vaccines and routine cervical cancer screening. Nevertheless, increased mortality in Japan due to this cancer may be a social problem. Although exact reason is not known, low coverage of the screening might be responsible for it.

HPV is a common virus that is transmitted horizontally through sexual contact [6], [7]. Approximately 80% of all women will become infected with HPV at some point in their lifetime, although about 90% of infections spontaneously regress within a few years [8]. Women who have persistent HPV infections develop cervical cancer [9]. To date, more than fifty HPV genotypes have been identified in the female reproductive tract, and certain HPV types cause invasive cervical cancer. Internationally, 13 HPV types, namely HPV 16, 18, 31, 33, 35, 39, 45, 51, 52, 56, 58, 59, and 68, are recognized as oncogenic or high-risk types (13HR) that can induce the cancer. In 2009, the World Health Organization (WHO) classified these 13HR types as 12 high-risk types: HPV 16, 18, 31, 33, 35, 45, 51, 52, 56, 58, and 59 (1A type), and one possible high-risk type, HPV68 (2A type).

Commercially available HPV tests, such as the hybrid capture generation 2 (HC2) and APTIMA tests target the 13HR HPV types, while the cobas HPV test does 14 high-risk types including the 13HR types and HPV 66 [10], [11]. However, some studies have suggested that other HPV types are associated with cancer development. Munoz et al. reported in an international survey that two additional types, HPV 73 and 82, should be considered carcinogenic [12]. An international surveillance study by de Sanjose et al. also reported that other HPV types, including HPV 26, 53, 66, 67, 69, 70, 73, and 82, have been identified in cervical cancer tissue by microdissection procedures using the INNO-Ripa test [13]. Moreover, Halec et al. conducted a systematic review of the biological evidence for carcinogenicity, demonstrating that HPV 26, 53, 66, 67, 68, 70, 73, and 82 may be oncogenic [14]. In Japanese studies, HPV types 67, 69, and 82 have been detected in invasive cervical cancer (ICC) [15], [16].

However, some recent reports have suggested that HPV test results are inconsistent among different assays [17], [18], [19], [20]. One of the most important issues is that some of the polymerase chain reaction (PCR)-based assays used in epidemiological studies have limitations. First, the highly sensitive PCR method detects not only the HPV type responsible for cancer but also the other HPV types co-existing the same specimen. Second, some PCR assays using consensus primers have shown inconsistent results in determining HPV types, as the sensitivity of detection is lower in some HPV types than in others. Even gold standard assays such as GP5+/GP6+ PCR, Roche LINEAR ARRAY and the INNO-Lipa which are widely used in many epidemiological study in Europe or the USA make no exception, since these assays are based with consensus primers. The consensus primer is designed to hybridize with many different HPV types. However, this type of system may have different efficacy for amplification of different HPV types, because of different numbers of mismatch between the primers and target sequences. Matsukura T has first demonstrated limitation of the consensus primers in GP5+/GP6+ PCR method [16]. In fact, Chan PK reported that GP5+/6+ exhibited a poor sensitivity for HPV52 [21]. Oštrbenk A reported that the cross reaction to HPV52 and HPV33, 35, and 58 causes a decrease of detection sensitivity of HPV52 in the Linear Array HPV Genotyping Test [20]. Lower sensitivity for HPV52 and cross-reactivity among HPV52, 33, 35, 58 may be critical demerit in analysis of Japanese cervical cancer, since HPV 52 and HPV58 are one of the most common HPV types in high-grade CIN (19) or cancer (15, 16).

Recently, one company established a multiplex PCR system called Genosearch-31 that can amplify 31 HPV types using a mixture of HPV type-specific primers, and detect each HPV type with HPV type sequence-specific oligonucleotide probe (SSOP) with the Luminex 100 xMAP flow cytometry dual-laser system (the PCR-SSOP-Luminex method). The sensitivity of the Genosearch HPV31 was confirmed to be equivalent (maximumly six time difference) for all target HPV types, with no cross-reactivity with each other type (19). Using this platform with a detection system for an additional 5 HPV types (Genosearch HPV31+5), we determined the HPV genotypes in pooled samples of 1526 cases of cervical intraepithelial neoplasia and 371 cases of invasive cervical cancer to elucidate probable high-risk HPV types in Japanese women.

## 2 Materials and methods

### 2.1 Subjects and sample preparation

This cross-sectional epidemiological study used liquid-based cytology (LBC) samples from cervical intraepithelial neoplasia (CIN), and LBC and tissue samples from invasive cervical cancer (ICC) obtained from Japanese women whose diagnoses were histologically confirmed. Written informed consent was obtained from all women for use of their HPV typing data, diagnoses, and ages. LBC samples were collected in the first visit in the outpatient clinics, and tissue samples were obtained from women at initial surgeries or at colposcopy examinations. Tiny tissue specimens were excised from cancer specimens by an experienced gynecological oncologist, and the remaining tissues were used for pathological diagnosis. Two clinical pathologists made diagnoses independently, and the final diagnosis was determined by consensus.

Details of the pooled samples are as follows. LBC samples of 936 CIN cases and 9 ICC cases were collected from multiple institutions from 2011 to 2012 (Japanese Human Papillomavirus Disease Education Research Survey [JHERS] study groups) (19), and of 621 CIN and 19 ICC cases were collected in outpatient clinic of Kanazawa Medical University Hospital from 2009 to 2017. Fresh tissue fragment samples from 211 ICC patients were collected from Hospitals of Kanazawa Medical University, Kanazawa University, and Osaka International Cancer Institute from 2002 to 2017, and formalin-fixed and paraffin-embedded tissue (FFPE) specimens from 141 ICC patients were collected from Kanazawa Medical University Hospital from 1990 to 2017. The pellet of 1 ml of LBC samples spin down by centrifugation, and a small fragment of tissue of cervical cancer specimen was stored at −80 °C until use.

DNA was extracted from the LBC samples and frozen tissue of ICC using the Smitest EX&D (MBL, Nagoya Japan), and from FFPE specimen using the pinpoint slide DNA isolation system (Zymo Research, Irvine, USA). DNA extracted from FFPE was immediately used for analysis.

A total of 31 CIN and 7 ICC samples were omitted due to poor quality for DNA. Ultimately, 1526 cases of CIN, including CIN1 (597 cases), CIN2 (526 cases), and CIN3 (403 cases), AIS (12 cases), and 371 ICC cases were examined (Table 1). ICC samples included squamous cell carcinoma (SCC), adenosquamous cell carcinoma (ADSCC), adenocarcinoma (ADC), and other histological types of cancer (OTC). The OTC included small- and large-cell neuroendocrine carcinoma and undifferentiated carcinoma.

**Table 1:** Human papillomavirus (HPV) genotypes detected as single or multiple infections in cervical intraepithelial neoplasia (CIN) and invasive cervical cancer (ICC).

	No. (%) of HPV positive specimens									
	All CIN	CIN1	CIN2	CIN3	AIS	ALL ICC	SCC	ADSCC	ADC	OTC
	N = 1526	N = 597	N = 526	N = 403	N = 12	N = 371	N = 279	N = 20	N = 59	N = 13
Any HPV types	1363(89.3)	516(86.4)	467(88.8)	380(94.3)	11(91.7)	337(90.8)	266(95.3)	19(95.0)	42(71.2)	10(76.9)
Single type infection	668(43.8)	217(36.3)	230(43.7)	221(54.8)	8(66.7)	299(81.5)	238(86.5)	17(85.0)	34(57.6)	10(76.9)
Multiple type infection	684(44.8)	298(49.9)	237(45.1)	149(37.0)	3(25)	31(8.4)	25(9.1)	1(5.0)	5(8.5)	0
Undetermined types	11(0.7)	1(0.2)	0	10(2.5)	0	7(1.9)	3(1.1)	1(5.0)	3(5.1)	0

This table shows the number and prevalence of HPV types detected in CIN, AIS, and ICC cases. “Any HPV types” indicates that any HPV types were detected. “Single type infection” indicates single-type infection. “Multiple type infection” indicates that multiple HPV types were detected. “Undetermined types” indicates that an unidentified HPV type was detected.

### 2.2 HPV genotyping and classification of HPV types

HPV genotyping was performed in a commercial laboratory (LSI, Tokyo, Japan). In brief, 10 ng of DNA was used in all analyses, and the beta-globin gene was used as internal control. Genosearch HPV31+ Genosearch HPV5 (GS-31, GS-5; Medical & Biological Laboratories Co., Ltd., Nagoya, Japan) was used with the PCR-SSOP-Luminex method. The GS-31 is a genotyping method that can detect 31 different HPV types, including 12 low-risk types (LRs), i.e., HPV 6, 11, 42, 44, 54, 55, 61, 62, 71, 84, 89, and 90; six probable high-risk types (Probable HRs), i.e., HPV 26, 53, 66, 70, 73, and 82; and the 13 internationally recognized high-risk types (13HRs), i.e., HPV 16, 18, 31, 33, 35, 39, 45, 51, 52, 56, 58, 59, and 68. The GS-5 was designed to detect an additional 5 probable HR types, i.e., HPV 30, 34, 67, 69, and 85. The Roche Linear Array detects 37 HPV types including 16 LRs, i.e., HPV 6, 11, 40, 42, 54, 55, 61, 62, 64, 71, 72, 81, 83, 84, IS39, and 89; eight probable HRs, i.e., HPV 26, 53, 66, 67, 69, 70, 73, and 82; and the 13HRs, i.e., HPV16, 18, 31, 33, 35, 39, 45, 51, 52, 56, 58, 59, and 68.

The Japanese Society of Obstetrics and Gynecology recommends dividing the 13HR types into two groups: relatively higher risk types (Relatively HR) and relatively lower risk types (Relatively LR). The former group includes HPV 16, 18, 31, 33, 35, 45, 52, and 58, and the latter includes HPV 39, 51, 56, 59, and 68. Based on this recommendation, the HR HPV types were further divided into four groups in the present study: HPV 16; HPV 18; high-grade HRs (HG-HR) HPV 31, 33, 35, 45, 52, and 58; and low-grade HRs (LG-HR) HPV 39, 51, 56, 59, and 68.

We ascertained that no LRs were identified as single types in high-grade squamous intraepithelial lesions (CIN3) and invasive cancer. Therefore, 10 ICC cases that showed infection with multiple HPV types, i.e., one HR and one or more LR HPV types, were conventionally classified as single-type infections of the HR type.

### 2.3 Statistical analyses

Chi-squared tests for trends were performed using GraphPad Prism ver. 6.05 (GraphPad Software, Inc., La Jolla, CA, USA).

## 3 Results

### 3.1 Common HPV types in cervical intraepithelial neoplasia (CIN) and invasive cervical cancer (ICC) in Japan

In cervical intraepithelial neoplasia (CIN), 1363 of 1526 (89.3%) cases were positive for HPV, and single- and multiple-type infections were detected in 43.8% (N = 668) and 44.8% (N = 684) of cases, respectively (Table 1). The prevalence of HPV infections did not differ among different CIN grades. However, the prevalence of single-type infections increased with increasing grade of CIN (chi-squared for trend, *P* < 0.0001), whereas that of multiple-type infection showed the opposite trend (chi-squared for trend, *P* < 0.0001). Thus, single-type infection was observed in 54.8% and 66.7% of CIN3 and adenocarcinoma in situ [AIS] cases, respectively, whereas it was observed in 36.3% of CIN1 cases. The single HPV types identified in CIN3 were 11 of the 13HRs, excluding HPV 45 and 59, and Probable HRs HPV 26, 66, 67, and 82 (Table 2). The top five HPV types identified in CIN3 were HPV 16, 52, 58, 51, and 31. Only HPV 16, 18, and 45 were detected in AIS (Table 2).

**Table 2:** HPV genotypes detected as single infections in CIN, AIS and ICC.

	CIN3	AIS	All ICC	SCC	ADSCC	ADC	OTC
	N = 230	N = 8	N = 306	N = 241	N = 18	N = 37	N = 10
HPV	No.(%)	No.(%)	No.(%)	No.(%)	No.(%)	No.(%)	No.(%)
16	91(39.6)	4(50.0)	149(48.7)	130(54.2)	4(22.2)	12(31.6)	3(30.0)
18	13(5.7)	3(37.5)	51(16.7)	21(8.8)	9(50.0)	17(44.7)	4(40.0)
26	1(0.4)	0	0	0	0	0	0
31	14(6.1)	0	15(4.9)	13(5.4)	0	0	2(20.0)
33	6(2.6)	0	3(1.0)	3(1.3)	0	0	0
35	1(0.4)	0	0	0	0	0	0
39	1(0.4)	0	2(0.7)	1(0.4)	0	1(2.6)	0
45	0	1(12.5)	4(1.3)	1(0.4)	1(5.6)	2(5.3)	0
51	15(6.5)	0	2(0.7)	2(0.8)	0	0	0
52	32(13.9)	0	36(11.8)	35(14.6)	1(5.6)	0	0
53	0	0	2(0.7)	1(0.4)	1(5.6)	0	0
56	3(1.3)	0	6(2.0)	6(2.5)	0	0	0
58	30(13.0)	0	12(3.9)	11(4.6)	0	0	1(10.0)
59	0	0	4(1.3)	3(1.3)	0	1(2.6)	0
66	2(0.9)	0	0	0	0	0	0
67	1(0.4)	0	3(1.0)	3(1.3)	0	0	0
68	6(2.6)	0	8(2.6)	7(2.9)	1(5.6)	0	0
69	0	0	1(0.3)	0	0	1(2.6)	0
70	0	0	1(0.3)	1(0.4)	0	0	0
73	0	0	0	0	0	0	0
82	4(1.7)	0	0	0	0	0	0
UK	10(4.3)	0	7(2.3)	3(1.3)	1(5.6)	3(7.9)	0

This table shows single-type HPV detected in CIN3, AIS, and ICC cases. They are arranged in order of HPV number. The gray area indicates the 13 internationally recognized high-risk (13HR) types, and the others are probable high-risk types. UK, unknown HPV type.

HPV types were examined in 371 ICC cases comprising 279 squamous cell carcinoma (SCC) cases, 20 adeno-squamous cell carcinoma (ADSCC) cases, 59 adenocarcinoma (ADC) cases, and 13 other histological type carcinoma (OTC) cases. A total of 90.8% of ICC, 95.3% of SCC, 95.0% of ADSCC, 71.2% of ADC, and 76.9% of OTC cases were positive for any type of HPV (Table 1). When only the cases infected with single-type HPV were analyzed, the prevalence was 81.5% in ICC cases, 86.5% in SCC, 85.0% in ADSCC, 57.6% in ADC, and 76.9% in OTC cases (Table 1). For common single-type HPV infections, 12 high-risk types (HRs), excluding HPV 35, and four Probable HRs, HPV 53, 67, 69, and 70, were identified in ICC cases (Table 2). The top five HPV types in ICC were HPV 16, 18, 52, 31, and 58 (Table 2), and those in SCC were HPV 16, 52, 18, 31, and 58 (Table 2). In order of prevalence, HPV 18, 16, and 45/52/53/68, and HPV 18, 16, 45, and 39/59/69 were detected in ADSCC and ADC, respectively (Table 2). These results may indicate that species alpha-9 HPV types (HPV 31, 52, and 58) were predominantly identified in SCC, whereas alpha-7 HPV types (HPV 39, 45, 59, and 68) were predominant in ADC. HPV 16 and 18 were commonly identified in both histological types, and both alpha-7 and alpha-9 HPV types were detected in ADSCC.

### 3.2 Distribution patterns of HR, Probable HR, and LR HPV types in CIN and ICC

When only single HPV type infections were evaluated, HR HPV types were detected in 78.0% (170/218) of CIN1, 82.2% (189/230) of CIN2, and 92.2% (213/231) of CIN3 cases. In contrast, only 16.1% of CIN1, 4.8% of CIN2, and none of CIN3 cases were positive for LR types.

**Fig. 1:**
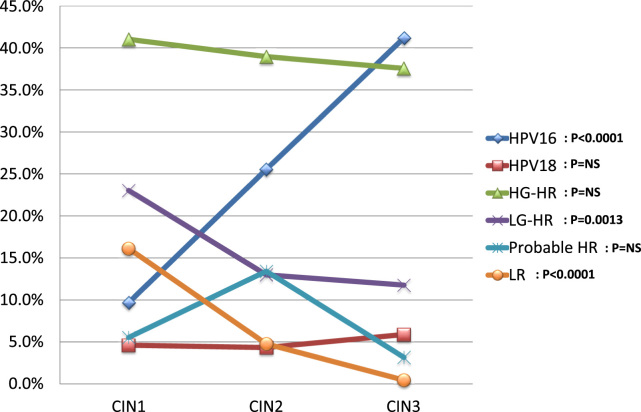
The prevalence of single-type HPV infections among different cervical intraepithelial neoplasia (CIN) grades. The prevalence of HPV types and groups are plotted according to CIN grade. Single-type infections of HPV 16, 18, high-grade high-risk (HG-HR), low-grade high-risk (LG-HR), probable high-risk (Probable HR), and low-risk (LR) groups were analyzed. HG-HR: HPV 31, 33, 35, 45, 52, and 58. LG-HR: HPV 39, 51, 56, 59, and 68. Probable HR: HPV 26, 53, 66, 67, 69, 70, 73, and 82. LR: HPV 6, 11, 42, 44, 54, 55, 61, 62, 71, 84, 89, and 90.

In the present study, HR HPV types are conventionally classified into the subgroups HPV 16, HPV 18, high-grade high-risk types (HG-HRs); HPV 31, 33, 35, 45, 52, and 58, and low-grade high-risk types (LG-HRs); HPV 39, 51, 56, 59, and 68. When only the cases with single-type HPV infection were evaluated, the prevalence of HR types (data not shown) and HPV 16 (chi-squared test for trend, *P* < 0.0001) increased with increasing grade of CIN (Fig. 1). In contrast, the prevalence of LG-HR HPVs (chi-squared test for trend, *P* = 0.0013) and LR types (chi-squared test for trend, *P* < 0.0001) significantly decreased with increasing CIN grade (Fig. 1). However, such differences were not observed at all for HPV 18, HG-HR, or Probable HR groups (Fig. 1).

**Fig. 2:**
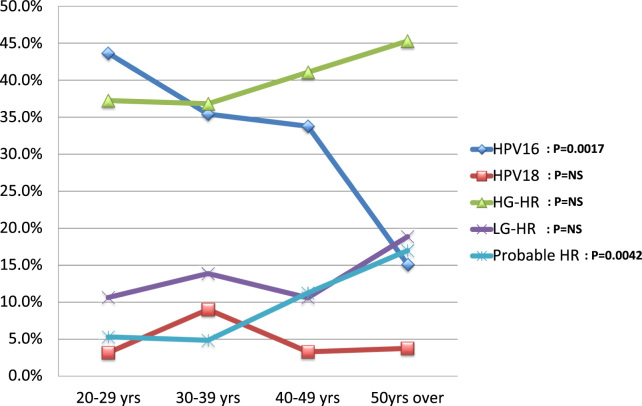
The age-dependent prevalence for single-type HPV infections groups in HSILs (CIN2 and CIN3). The prevalence of HPV types and groups are plotted according to patient age. Single-type infections of HPV 16, 18, high-grade high-risk (HG-HR), low-grade high-risk (LG-HR), probable high-risk (Probable HR), and low-risk (LR) groups were analyzed. HG-HR: HPV 31, 33, 35, 45, 52, and 58. LG-HR: HPV 39, 51, 56, 59, and 68. Probable HR: HPV 26, 53, 66, 67, 69, 70, 73, and 82. LR: HPV 6, 11, 42, 44, 54, 55, 61, 62, 71, 84, 89, and 90.

When age-dependent prevalence for single-type HPV infection groups in CIN2 and CIN3 were evaluated, the prevalence of HPV 16 significantly decreased with increasing patient age (chi-squared test for trend, *P* = 0.0017), whereas Probable HR showed the opposite trend (chi-squared test for trend, *P* = 0.0042) (Fig. 2). No such tendencies were observed for HPV 18, HG-HR, or LG-HR groups (Fig. 2). No single-type LRs were identified in HSILs.

**Fig. 3:**
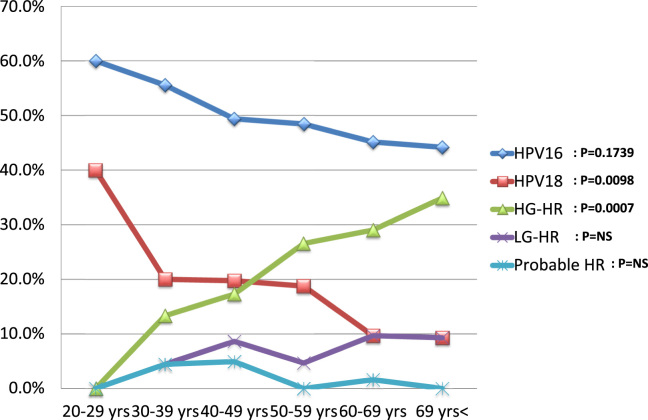
The age-dependent prevalence for single-type HPV infections groups in invasive cervical cancer (ICC). The prevalence of HPV types and groups are plotted according to patient age. Single-type infections of HPV 16, 18, high-grade high-risk (HG-HR), low-grade high-risk (LG-HR), and probable high-risk (Probable HR) groups were analyzed. HG-HR: HPV 31, 33, 35, 45, 52, and 58. LG-HR: HPV 39, 51, 56, 59, and 68. Probable HR: HPV 26, 53, 66, 67, 69, 70, 73, and 82.

**Fig. 4:**
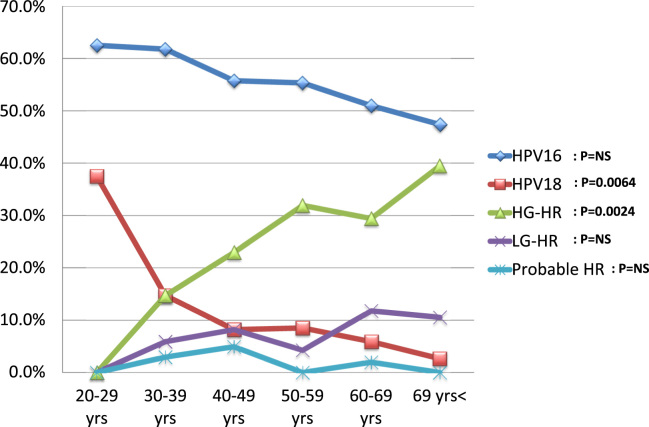
The age-dependent prevalence for single-type HPV infections groups in squamous cell carcinoma (SCC). The prevalence of HPV types and groups are plotted according to patient age. Single-type infections of HPV 16, 18, high-grade high-risk (HG-HR), low-grade high-risk (LG-HR), and probable high-risk (Probable HR) groups were analyzed. HG-HR: HPV 31, 33, 35, 45, 52, and 58. LG-HR: HPV 39, 51, 56, 59, and 68. Probable HR: HPV 26, 53, 66, 67, 69, 70, 73, and 82.

When age-dependent prevalence for single-type infection was examined in ICC cases, the prevalence of HPV 18 (chi-squared test for trend, *P* = 0.0098) and HPV16 (chi-squared test for trend, *P* = 0.1793) decreased with increasing patient age (Fig. 3), although that for HPV16 was not significant. In contrast, the prevalence of HG-HR types increased with patient age (chi-squared test for trend, *P* = 0.0007). Such tendencies were not seen in LG-HR, or Probable HR HPV types. The same analysis was done for SCC, ADSCC, and ADC cases, and only SCC exhibited a similar trend to ICC. It should be noted that all patients with SCC and ICC aged 20–29 years were positive for either HPV 16 or HPV 18 (Fig. 3, Fig. 4).

## 4 Discussion

### 4.1 Potential high-risk HPV types for cervical cancer in Japan

To elucidate potential high-risk (oncogenic) HPV types in Japanese women, we identified HPV types infecting HSIL (CIN2 and 3) and ICC cases using a new HPV genotyping test that can detect 36 HPV types including 13 h, 11 Probable HRs, and 16 LRs. The present results demonstrated that 11 of the 13HRs (excluding HPV 45 and 59) and probable HR types such as HPV 26, 66, 67, and 82, were detected as single HPV types in CIN3, and 12 of 13 h HPV types (excluding HPV 35) and Probable HR types such as HPV 53, 67, 69, and 70 were identified in ICC. It is noted that HRs HPV 39 and 59 and the Probable HRs HPV 53 and 67 were detected as single-type infections at the same prevalence in ICC, and HPV 35 and HPV 26, 66, 67, and 82 had the same prevalence in CIN3. These results suggest that HPV 26, 53, 66, 67, 69, 70, and 82 are likely to be oncogenic types. In 2012, the IARC working group defined 12 high-risk types (group 1A) and the following additional types as probable (group 2A), or possible (group 2B) [22]. Group 1A includes the 12 internationally defined high-risk types, including HPV 16, 18, 31, 33, 35, 39, 45, 51, 52, 56, 58, and 59; the 2A group contains HPV 68; and the 2B group contains HPV 26, 53, 66, 67, 70, 73, and 82. Halec et al. demonstrated that eight HPV types (HPV 26, 53, 66, 67, 68, 70, 73, and 82) have been rarely but consistently identified as single HPV infections in about 3% of cervical cancer tissues, and showed that the biological behavior of these types is similar to that of carcinogen 1A types [14]. In the present study, most of the Probable HRs (except HPV 73) were identified in HSIL or ICC, suggesting that Probable HRs may potentially be HRs in Japan.

### 4.2 Distribution patterns of HPV types in CIN and cervical cancer

When only single-type infection was evaluated, the present study demonstrated that LR types were observed in CIN1 and CIN2, whereas they were not identified in CIN3 and ICC. In contrast, HR types and Probably HR types were identified in CIN3 and ICC. An evaluation of single type infections estimates the risk of each HPV type for cancer.

The same analysis showed that HPV 31, 33, 52, 58 (species alpha-9), 51 (species alpha-5), and 56 (species alpha-6) were identified in SCC, whereas these types were not identified in adenocarcinoma (ADC). In contrast, HPV 18 and 45 (species alpha-7) were more common in ADC than in SCC. The former types were predominantly the alpha-9, and the latter types were the alpha-7. Moreover, HPV 45 was not identified as a single type at all in CIN3, whereas it was identified in AIS. These data suggest that HPV types in the alpha-9 predominantly induce SCC, whereas HPV types belonging to the alpha-7 induce ADC. However, HPV 16 (the alpha-9) and 18 (The alpha-7) appear to induce both types of cancer. Previous studies demonstrated that HPV 16, 18, 45, and 52 are common HPV types in ADC [23], [24]. The present data showed HPV 52 was identified in one case of ADSCC, but not in ADC (Table 2B).

### 4.3 CIN-grade or age-dependent distribution of HPV type in CIN and ICC patients

Many reports suggest that HPV 16 and 18 are the most malignant HPV types, as their progression from premalignant to malignant lesions is earlier than that of other types [25], [26], [27], [28], [29], [30]. To estimate malignant potential, the trend test for CIN grade and patient age was performed. Among HRs, HPV 16 increased significantly with increased CIN grade, whereas LG-HR types exhibited opposite trends. Surprisingly, such trends were not seen in HPV 18 or HG-HRs (Fig. 1). This may suggest that only HPV 16 has a strong potential for CIN progression, whereas the other HR and LR types do not.

In the analysis of age-dependent prevalence in SCC, HPV 18 significantly decreased with increased age of patients. Such trend for HPV16 was not significant. In contrast, the opposite trend was observed in HG-HR types (HPV 31, 33, 35, 45, 52, and 58). HPV18 appeared to induce SCC more quickly than

other HR types did if the infecting age was the same. These results may support the previous finding that HPV 16 and 18 are the most aggressive in promoting malignant progression in squamous cells [26], [27], [28], [30]. It should be noted that all ICC cases aged 20−29 were positive for HPV 16 or 18 in the present study, suggesting that currently available prophylactic HPV vaccines may be extremely effective for preventing ICC in young Japanese women. This insight may provide important issue to Japanese nation in HPV vaccination crisis [31]. On the other hand, the present analysis demonstrated that the behavior of HPV 16 and 18 is not exactly the same. The prevalence of HPV 16 increased with increased grade of CIN, whereas that of HPV 18 did not. Moreover, the prevalence of HPV 18 significantly decreased with patient age, while it was not significant for HPV16 in SCC. Such differences may suggest different mechanisms in the induction of SCC by these two types. The increasing prevalence of HPV 16 according to higher grades of CIN may suggest that HPV 16-infected cells are segregated to develop CIN3. In contrast, such tendency was not observed for HPV 18, along with the higher prevalence of HPV 18 in SCC in younger women. These results suggest that HPV18 may induce SCC through the *de novo* pathway, since the cancer appears to be rapidly developed by HPV18 than other high-risk HPV types. Some studies have suggested that CIN3 may be developed directly from normal epithelium [32], [33], [34]. This hypothesis of *de novo* carcinogenesis by HPV18 warrants further investigation in the future study.

### 4.4 Limitation

Although Genosearch-31 was used in the previous epidemiological study (19), Genosearch-31 plus 5 was first used in the present study. The limitation of this study may be lack of the data of comparative study with the gold standard methods such as Roche LINEAR ARRAY and the INNO-Lipa.

## 5 Conclusions

We elucidated the prevalent HPV types in CIN3 and ICC in Japanese women using a new HPV genotyping method in evaluation of single-type HPV infections. Probable HRs such as HPV 53, 67, 69, 70, and 82 were also identified as single-type infections as equivalent prevalence as the internationally established HR HPV types in CIN3 and ICC. This suggests that these types are likely to be HR types in cervical cancer in Japanese women. HPV 16 and 18 can induce both SCC and ADC. Age or CIN grade -dependent distribution suggest that HPV18 induces cervical cancer rapidly, and the progression might be through de novo pathway. HPV 31, 52, and 58 (species alpha-9) predominantly induced high-grade squamous lesions and SCC, whereas HPV 39, 45, and 59 (species alpha-7) induced ADC. All ICC cases aged 20–29 were positive for HPV 16 or 18, suggesting that currently available prophylactic HPV vaccines may be quite effective on prevention of ICC in young Japanese women.

## Conflicts of interest

These authors declare no potential conflicts of interest.
